# Feasibility and Clinical Relevance of a Mobile Intervention Using TrackPAD to Support Supervised Exercise Therapy in Patients With Peripheral Arterial Disease: Study Protocol for a Randomized Controlled Pilot Trial

**DOI:** 10.2196/13651

**Published:** 2019-06-26

**Authors:** Katrin Paldán, Jan Simanovski, Greta Ullrich, Martin Steinmetz, Christos Rammos, Rolf Alexander Jánosi, Susanne Moebus, Tienush Rassaf, Julia Lortz

**Affiliations:** 1 Centre for Urban Epidemiology Institute for Medical Informatics, Biometry und Epidemiology University of Duisburg-Essen Essen Germany; 2 Centre of Competence Personal Analytics at the University of Duisburg-Essen Department of Engineering Sciences University of Duisburg-Essen Duisburg Germany; 3 Department of Cardiology and Vascular Medicine West-German Heart and Vascular Center Essen University of Duisburg-Essen Essen Germany

**Keywords:** peripheral arterial disease, telemedicine, patient participation, patient compliance, primary health care

## Abstract

**Background:**

Peripheral arterial disease (PAD) is a common and severe disease with a highly increased cardiovascular morbidity and mortality. Through the circulatory disorder and the linked undersupply of oxygen carriers in the lower limbs, the ongoing decrease of the pain-free walking distance occurs with a significant reduction in patients’ quality of life. Studies including activity monitoring for patients with PAD are rare and digital support to increase activity via mobile health technologies is mainly targeted at patients with cardiovascular disease in general. The special requirement of patients with PAD is the need to reach a certain pain level to improve the pain-free walking distance. Unfortunately, both poor adherence and availability of institutional resources are major problems in patient-centered care.

**Objective:**

The objective of this trackPAD pilot study is to evaluate the feasibility of a mobile phone–based self tracking app to promote physical activity and supervised exercise therapy (SET) in particular. We also aim for a subsequent patient centered adjustment of the app prototype based on the results of the app evaluation and process evaluation.

**Methods:**

This study was designed as a closed user group trial, with assessors blinded, and parallel group study with face-to-face components for assessment with a follow-up of 3 months. Patients with symptomatic PAD (Fontaine stage IIa or IIb) and possession of a mobile phone were eligible. Eligible participants were randomly assigned into study and control group, stratified by their distance covered in the 6-min walk test, using the software TENALEA. Participants randomized to the study group received usual care and the mobile intervention (trackPAD) for the follow-up period of 3 months, whereas participants randomized to the control group received only usual care. TrackPAD records the frequency and duration of training sessions and pain level using manual user input. Clinical outcome data were collected at the baseline and after 3 months via validated tools (6-min walk test, ankle-brachial index, and duplex ultrasound at the lower arteries) and self-reported quality of life. Usability and quality of the app was determined using the user version of the Mobile Application Rating Scale.

**Results:**

The study enrolled 45 participants with symptomatic PAD (44% male). Of these participants, 21 (47%) were randomized to the study group and 24 (53%) were randomized to the control group. The distance walked in the 6-min walk test was comparable in both groups at baseline (study group: mean 368.1m [SD 77.6] vs control group: mean 394.6m [SD 100.6]).

**Conclusions:**

This is the first trial to test a mobile intervention called trackPAD that was designed especially for patients with PAD. Its results will provide important insights in terms of feasibility, effectiveness, and patient preferences of an app-based mobile intervention supporting SET for the conservative treatment of PAD.

**International Registered Report Identifier (IRRID):**

DERR1-10.2196/13651

## Introduction

### Background

Peripheral arterial disease (PAD) is a common atherosclerotic disease affecting the lower extremities. The prevalence of PAD is high as almost one-fifth of the population aged 65 years or above in the high-income countries is diseased and the occurrence increases further with age [1]. Right after coronary arterial disease and the cerebrovascular arterial disease, PAD is the third most common atherosclerotic disease [1,2]. However, PAD does not only limit an active lifestyle with the risk for lower limb amputation, but it is also an independent predictor of cardiovascular morbidity and mortality [3,4]. Limitations in daily life often arise from intermittent claudication (IC), which is defined as an impairment of walking because of pain, tiredness, or discomfort in the legs during walking and is relieved by rest. IC is a common and debilitating symptom of PAD and is also associated with a significant reduction in patients’ quality of life [5]. The pain-free walking distance decreases with further disease progression. In addition to this symptom and a significant lower quality of life, the most dreaded complication in PAD is the loss of the affected extremity.

Supervised exercise therapy (SET) is one of the most effective options in the conservative management of PAD [6]. Through a complex mechanism that includes arteriolar dilation, changes in microcirculation and endothelial function without directly improving limb blood flow, SET was shown to improve the pain-free walking distance and also the quality of life [7-11]. However, 2 challenges in the conservative management of patients with PAD arise. First, the availability of institutional resources for SET is rare. PAD patients are undersupplied in care, compared with patients with coronary artery disease [12,13]. Second, the adherence to guideline recommendation regarding physical training is rather low [14-16].

Studies including activity monitoring for PAD patients are rare and are mainly focusing on the overall activity, neglecting the training to the pain threshold as required for SET [16-18], although SET was shown to have more beneficial effects than the simple increase in activity. This fact may be responsible for the conflicting results in past studies. Another limiting factor in previous studies was the use of (telephone) counseling, which relativizes the effect of reduced personnel deployment.

With the use of mobile health (mHealth) technologies, we see the potential for a wider accessibility without an excessive increase of personnel resources. In particular, with an increasing focus on personalized mHealth, highlighting health education and changing people's health-related behavior [19-21], mHealth technologies have the potential to solve the current problems of missing adherence and infrastructure. Patients with PAD deserve more attention regarding their therapeutic options as their outcome and the guideline adherence of their treating physicians is still poor [22].

We developed trackPAD (Rocket Apes GmbH) as the first app-prototype for patients with PAD that should increase patients’ empowerment and improve their care. TrackPAD should support patients to implement SET in everyday life. It is also thought to overcome motivational barriers leading to a higher adherence to training instructions and therefore aiming for a slower disease progression. TrackPAD might also have the potential to (partly) compensate the missing infrastructure, such as training or support groups, by sharing personal success and competing against each other.

The overall aim for the implementation of trackPAD is to close supply gaps in care and provide digital solutions for patients with PAD to overcome personal and structural barriers by reaching a wide availability and high cost-effectiveness at the same time.

We will evaluate the potential benefits of mHealth‑based SET performance to reduce disease progression and test the feasibility of the developed mobile phone app. The following app evaluation will also give important insights for the patient-centered app development in this special patient collective.

### Objective

The aim of this pilot study was to evaluate the clinical relevance and the feasibility of an app to support SET in patients with PAD.

## Methods

### Research Questions

The trackPAD pilot study aims to answer the following questions:

1. Is trackPAD suitable for recording the patient's daily/weekly walking distance and the quality of performed SET (units)?
2. Is the app suitable for the target group and for further study purposes?
3. Does the use of the app increase physical activity and performance of SET resulting in an improvement of patients’ 6-min walk test distance?
4. Is the app feasible to implement in everyday practice?

To address research question 1, 2, and 4, we asked the patients after 3 months on how they evaluated the app, assessed reasons for dropout in detail, and analyzed the log file data of the app.

To address research question 3, we analyzed whether patients directed by trackPAD showed an increase in the 6-min walk test distance after 3 months. A minimal clinical increase of 20m was already shown to be beneficial in patients with IC [17,23]. The results were compared with a control group, which did not use trackPAD during the study period.

### Measures

To evaluate the feasibility of the app, a questionnaire survey regarding the trackPAD evaluation will be performed at follow-up based on an already standardized instrument for app evaluation [24].

The questionnaire, slightly adapted and shortened for the study objectives, comprises 5 parts:

- Functionality
- Aesthetics
- Information
- Overall evaluation of subjective app quality
- Perceived effects regarding the implementation of structured walking training/SET

In addition, a detailed description of the log file data of the patients (Table 1) and the verbally reported reasons for not using trackPAD over 3 months will be given. To evaluate the clinical relevance of the app, we will evaluate the following outcomes variables.

**Table 1:** Relevant accessible data of trackPAD participants’ use in real time.

Accessible data	Subcategories
Technical information	Mobile phone operating system and version; personal trial number (anonymous)
Overall use	Number of medals won; frequency and duration of use (not exercise!)
Summary	Total length of all SET^a^ units; number of steps; number of performed SET units; breaks (number+duration)
Weekly overview	Number of chosen SET units; frequency and duration of SET units and intervals; number of steps; number of performed SET units; number of performed SET units in relation to previously set weekly goal (less or more than initially aimed for); increase of performed SET units compared with previous week
Pro-SET unit	Length of SET unit and time (date and time); number of performed steps; number of intervals needed to finish SET unit
Prointerval	Evaluation of SET unit (pain, breath, overall intensity); length of the interval; number of performed steps

^a^SET: supervised exercise therapy.

### Outcome Variables

#### Primary Outcome

The primary outcome was defined as the change in pain-free walking distance and was assessed by comparing the meters covered in a 6‑min walk test using a standardized protocol [25] at baseline and after the 3 months follow-up. The 6-min walk test was performed under the supervision of a trained exercise technician. Participants were instructed to cover as much distance as possible and walk up and down a 50-m hallway for up to 6 min. Participants were instructed to push a measuring wheel along for the full 6 min of the test, but were allowed to take breaks if necessary. They were also allowed to use an assistive device during both the walking tests if they so desired. The technician stood in the middle of the course and supervised the walking test, but did not encourage participants. The total distance walked in the test was read off the measuring wheel.

#### Secondary Outcome

Secondary outcome measures were any changes in perfusion indices, including ankle-brachial index (ABI) at rest or after treadmill test (3.0 km/h and incline of a 10% slope), according to the current European Society of Cardiology (ESC) guidelines on the diagnosis and treatment of PAD [26].

In addition, changes in the large elastic arterial stiffness determined through pulse wave velocity were recorded. Noninvasive duplex ultrasound was performed at the lower leg arteries (Arteria tibialis posterior and anterior). Standard techniques, as determined by Doppler and duplex ultrasonography, were used to quantify tissue perfusion.

Further assessment of quality of life and subjective physical activity should capture potential benefits resulting from improvement of activity. PAD-specific quality of life was determined through the PADQOL, a validated PAD-specific quality of life questionnaire [27].

### Missing Data/Data Cleaning

Multiple imputation missing data handling procedures [28] were implemented using multivariate imputation by chained equations [29], a package for the R statistical software environment (The R foundation, version 3.5.0). As a last resolve, all missing data values in the final dataset were multiple imputed according to methodology suggested by Schafer and Graham [30] and Barnes et al [31].

### Study Design and Inclusion Criteria

The trackPAD pilot study was designed as a 2-armed randomized controlled trial and included patients with diagnosed and symptomatic PAD. This was a closed user group trial, with assessors blinded, and parallel group study with face-to-face components for assessment with a follow-up of 3 months. The participants were randomly assigned and stratified by their walking distance to control and study group after giving their written informed consent. The study procedure required 2 visits at the vascular outpatient clinic of the University Clinic of Essen at baseline (November 2018 to mid-December 2018) and 1 additional at follow-up (expected during early February 2019 to mid-March 2019). The first visit at baseline included all clinical pretestings and the quality of life questionnaires. The second baseline visit included a 10-min lecture repeating instructions for SET. After the lecture, participants received the result of the randomization, their group assignment, and the study group remained for downloading the app and a short guide to the app. The third visit was the follow-up visit at the end of the study.

No further visits during the study periods were planned. Nevertheless, in case of technical issues (such as system failures and bugs), technical support was offered to the participants, which was operated by nonmedical personnel. The support was reachable by phone (hotline) or email. All emails were answered within the next 24 hours and the hotline was operated from Mondays to Fridays (for 4 hours) between 8 and 12 pm. During the entire study period, the software engineers of trackPAD were available to fix any bugs or technical events that had occurred. Therefore, we were able to deliver necessary updates to the participants. For an update (ie, not working step counter), we contacted the participants and provided a written manual and oral instructions. For further needs, we additionally offered face-to-face appointments that took place at the vascular outpatient clinic and were provided by nonmedical personnel. As we planned, the provided updates did not change the use, behavior, or any feature of trackPAD; they only fixed bugs and technical issues in the code.

The inclusion criteria included patients with diagnosed and symptomatic PAD in the lower extremities, who were aged at least 18 years. PAD diagnosis had to be based on at least one of the following criteria: (1) ABI of 0.9 or less in at least one leg [32], (2) invasive or noninvasive imaging of stenotic lower extremity artery disease, or (3) endovascular or surgical revascularization of a lower extremity artery. Symptomatic PAD in the lower extremity had to be also characterized by Fontaine stage II (IC after walking). In addition, the possession of a mobile phone was obligatory (mobile phone with iOS 11.0 or later or Android 5.0 or later, suitable for downloading trackPAD). Giving the written informed consent before any study procedure was mandatory.

The following exclusion criteria were defined: acute or critical limb ischemia, severe angina pectoris (by Canadian Cardiovascular Society score 3-4), myocardial infarction/stroke in the last 3 months, active congestive heart failure requiring the initiation or uptitration of diuretic therapy, congestive heart failure with severe symptoms (by New York Heart Association score 3-4), active arrhythmia requiring the initiation or uptitration of antiarrhythmic therapy, severe valve disease, active cancer or malignancy, severe cognitive dysfunction (defined as dementia), leg pain at rest (Fontaine stage III or IV), no German language knowledge, walking impairment because of other causes than PAD, below or above knee amputation, wheelchair bound and/or use of a walking aid. Textbox 1 summarizes the inclusion and exclusion criteria.

**Inclusion and exclusion criteria of the trackPAD pilot study.**

Inclusion criteria

- Age ≥18 years
- Diagnosis of lower extremity peripheral arterial disease (PAD) based on any of the following:
  - Ankle-brachial index ≤0.9 in at least one leg
  - Invasive or noninvasive imaging of stenotic lower extremity artery disease
  - Endovascular or surgical revascularization of lower extremity artery
- PAD Fontaine stage IIa—mild claudication
- PAD Fontaine stage IIa—moderate-severe claudication
- Mobile phone with possibility to use trackPAD:
  - Android 5.0 or later
  - iOS 11.0 or later
- Written informed consent before any study procedures, including a specified follow-up evaluation
- Best medical treatment in the last 2 months in accordance with standard guidelines

Exclusion criteria

- Wheelchair bound, use of walking aid, or walking impairment because of other causes than PAD
- Below or above knee amputation
- PAD Fontaine stage I—asymptomatic
- PAD Fontaine stage III—ischemic rest pain
- PAD Fontaine stage IV—ulceration or gangrene
- Acute or critical limb ischemia
- Severe angina pectoris according to Canadian Cardiovascular Society class (score 3-4), or myocardial infarction, or stroke in the last 3 months
- Active congestive heart failure requiring the initiation or uptitration of diuretic therapy
- Severe congestive heart failure according to New York Heart Association (score 3-4)
- Active arrhythmia requiring the initiation or uptitration of antiarrhythmic therapy
- Severe valve disease
- Active cancer or malignancy
- Severe cognitive dysfunction
- No German language knowledge

### Recruitment and Randomization

Information regarding the pilot study and a call for participation were announced in a local newspaper (Westdeutsche Allgemeine Zeitung, local section for Essen and Duisburg) with contact information provided, including phone number and email address (trackPAD@uk-essen.de). Further potential participants were actively asked during their visits to the outpatient clinics or during their inpatient stay in the Department of Cardiology and Vascular Medicine, University Clinic of Essen. Interested patients with known PAD were asked to fill out a questionnaire exclusively developed for our study purpose. The questionnaire included questions about the patients’ social background, the knowledge about SET, the personal health status, and the possession of a mobile phone. The questionnaire ended asking whether the patient was willing to participate in the trackPAD pilot study. As the questionnaire was anonymous, willing patients were asked to register for the upcoming pilot trail at the front desk of the outpatient clinic.

After screening in terms of inclusion and exclusion criteria of suitable participants and obtaining written informed consent from each participant, they were randomized by the Center for Clinical Studies in Essen using the TENALEA software into 2 groups. The control group included participants with standard care and no further mobile intervention. The study group included participants with standard care and additional mHealth-based self-tracking of their physical activity using trackPAD. The participants were stratified by their results during the 6‑min walk test (distance lesser than 362m, between 362m and 430m, and greater than 430m). After the randomization process, no participants, regardless of the reason for exclusion, were replaced.

All participants were invited to a lecture (10 min) repeating the instructions for SET and handing out a flyer summarizing SET execution. After the lecture, participants received the result of the randomization and their group assignment. The study group remained for downloading the app and a short guide to the app. Technical issues were resolved immediately after installation by nonmedical personnel. To prevent any bias, based on disappointment or lack of motivation because of allocation into the control group, the allocation to the groups were announced only after the presentation and not before this event. All participants that did not show up to the introduction were contacted and scheduled for a new appointment within the next week. The presentation was demonstrated separately to everyone not present and the flyer was also handed out to each participant.

### Baseline

Study and control group received the same baseline examinations during their first visit. Clinical measurements including 6‑min walk test, ABI at rest and after physical activity, and pulse wave measurement were obtained. A blood sample was also taken to record the levels of various parameters, including total cholesterol, low-density lipoprotein and high-density lipoprotein, and triglycerides. In addition, participants’ demographics and past medical history were documented. The assessors of the clinical outcomes were blinded regarding participants’ randomization to the study or control group. During their first medical visit, both groups received the instruction to perform SET according to the current standard guidelines that recommend 3 units weekly for 30 to 60 min [26], but patients were kept open about how often they performed SET. The guidance in terms of SET included an oral recommendation and instruction by the same treating physician for all participants. In addition, all participants received a flyer with a summary of important information for SET, including the guideline recommendation of 3 units weekly for 30 to 60 min. The first baseline visit included a structured interview. The interview was conducted by medical personnel. A questionnaire served as structured guideline, which was used for all patients at baseline and follow-up. The questionnaire included personal data, questions on quality of life and PAD-specific quality of life [27], health status, and lifestyle-related questions (physical activity and smoking).

### Follow-Up

The planned study duration was 3 months and the completion of the follow-up was planned for the end of April 2019. All participants received the follow-up examinations, including a retake of all previously performed clinical examinations and completion of the questionnaire on secondary outcomes. Moreover, any changes in personal medical history or medication since baseline were recorded. Similar to at baseline, the assessors of the clinical outcomes were blinded. Quality of life and PAD-specific quality of life [27], health status, and lifestyle-related questions were asked again in a structured interview by medical personnel. To evaluate the feasibility of the app in the study group, an additional questionnaire survey regarding the trackPAD evaluation was performed at follow-up, based on an already standardized instrument for app evaluation with slight adaption for study purposes [24]. In addition, a detailed description of the log file data of the patients (Table 1) and the verbally reported reasons for not using trackPAD over 3 months were given.

### TrackPAD

For this pilot study an exclusively developed mHealth-based app (trackPAD) was used to track patients’ physical activity during the study period. TrackPAD was thought to represent the first mobile intervention to support patients with PAD regarding their implementation of SET. As mobile interventions lack in general the possibility of a direct measurement of onset and extent of claudication, we assessed breaks within each SET unit that were rated by the users in terms of pain level, breathing, and overall exhaustion before resumption of the SET unit. Through the detection of number and duration of breaks within a SET unit and the subjective pain assessment over time, we were able to detect changes in SET performance.

**Figure 1:**
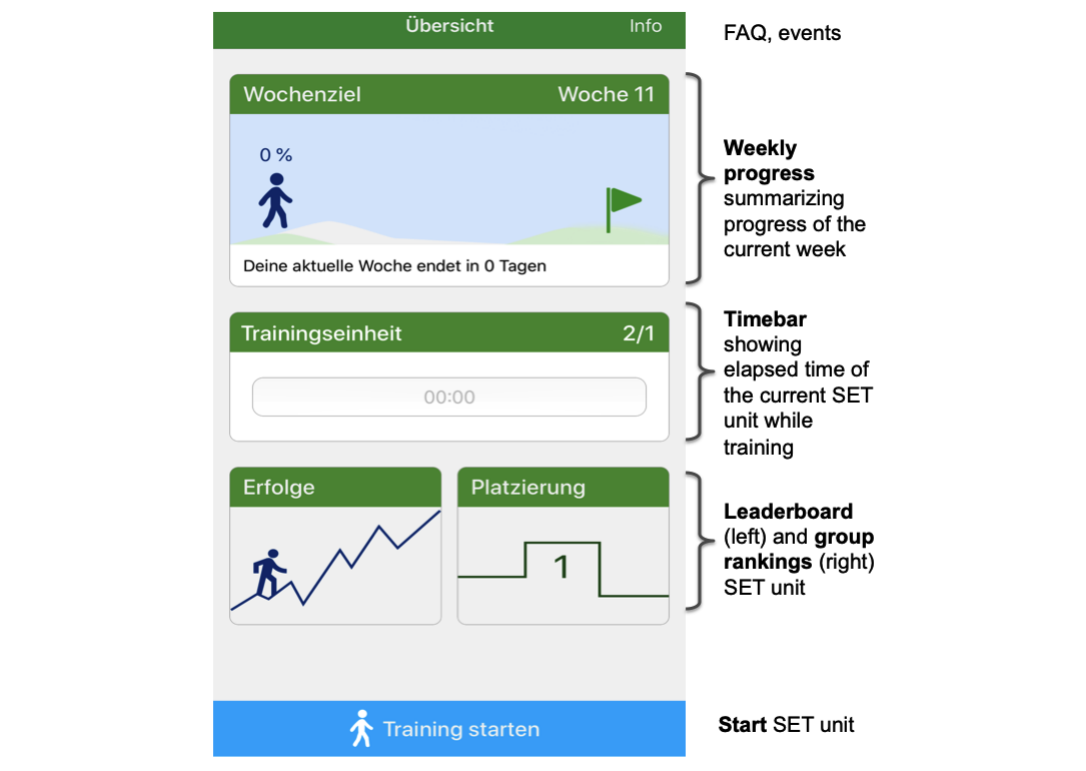
Main view of trackPAD. Weekly progress overview (upper part) and time bar active while training (central part). The main view also offers the possibility to access personal achievements (lower left part) or the leaderboard (lower right part). FAQ: frequently asked questions; SET: supervised exercise training.

Patients’ physical activity is tracked after actively starting a SET unit using the start button within trackPAD. No assessment of the background activity is performed and patients have to start their training actively. TrackPAD records the frequency and duration of training sessions and pain level using manual user input. The time bar in the main screen (Figure 1) indicates the minutes of exercise already performed during the SET unit. Each unit can be paused or stopped. After pausing and before the resumption of the SET unit, patients have to rate their pain level, breathing, and overall exhaustion (weekly goal and self-evaluation of the training).

**Figure 2:**
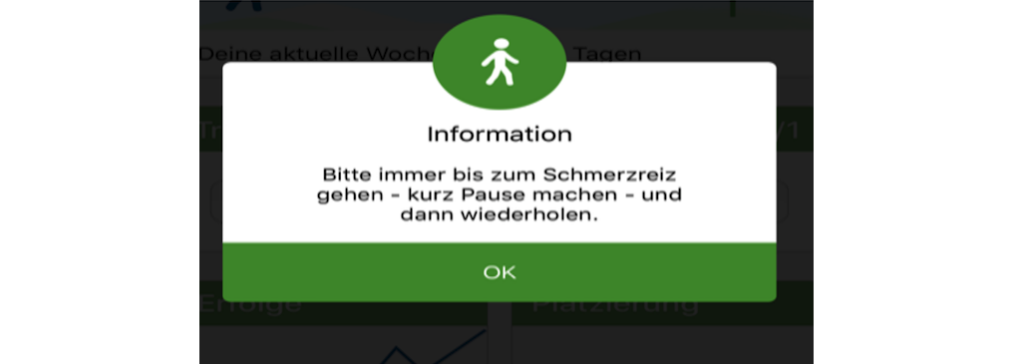
Claudication reminder. After starting each supervised exercise training (SET) unit a pop-up appears reminding that a certain claudication level should be reached following a short break and repetition. The pop-up needs to be actively confirmed to begin the SET.

**Figure 3:**
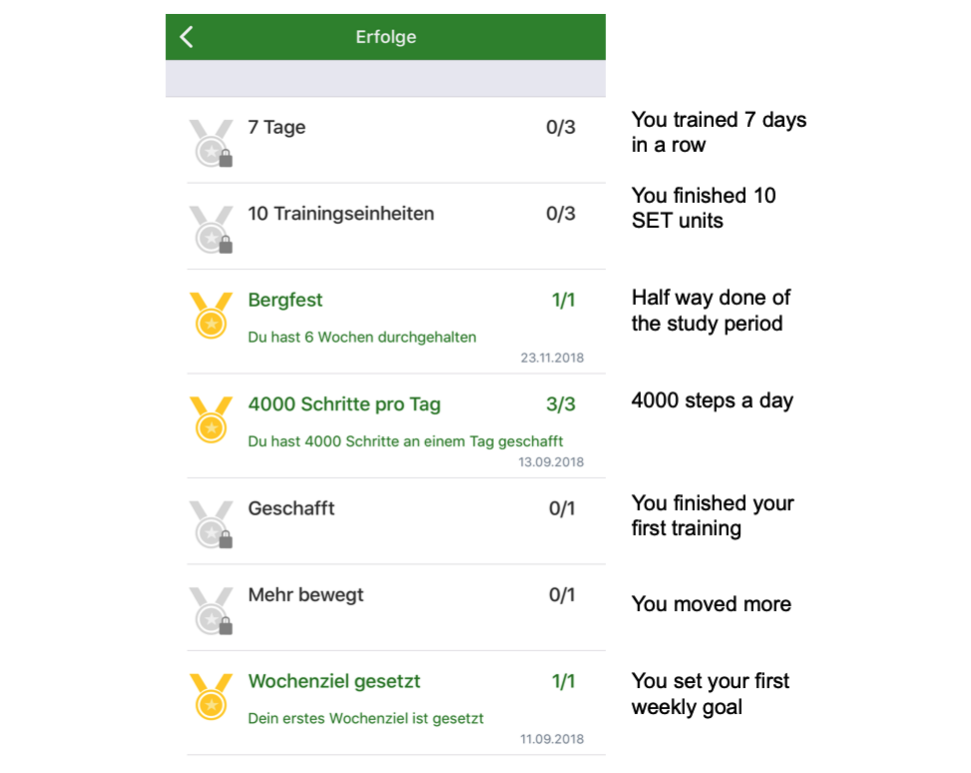
Personal achievements page. Reaching personal achievements unlocks medals in the medal mirror. The numbers at the right indicate the number of possible medals to unlock (eg, gold medal, silver medal, bronze medal). SET: supervised exercise training.

**Figure 4:**
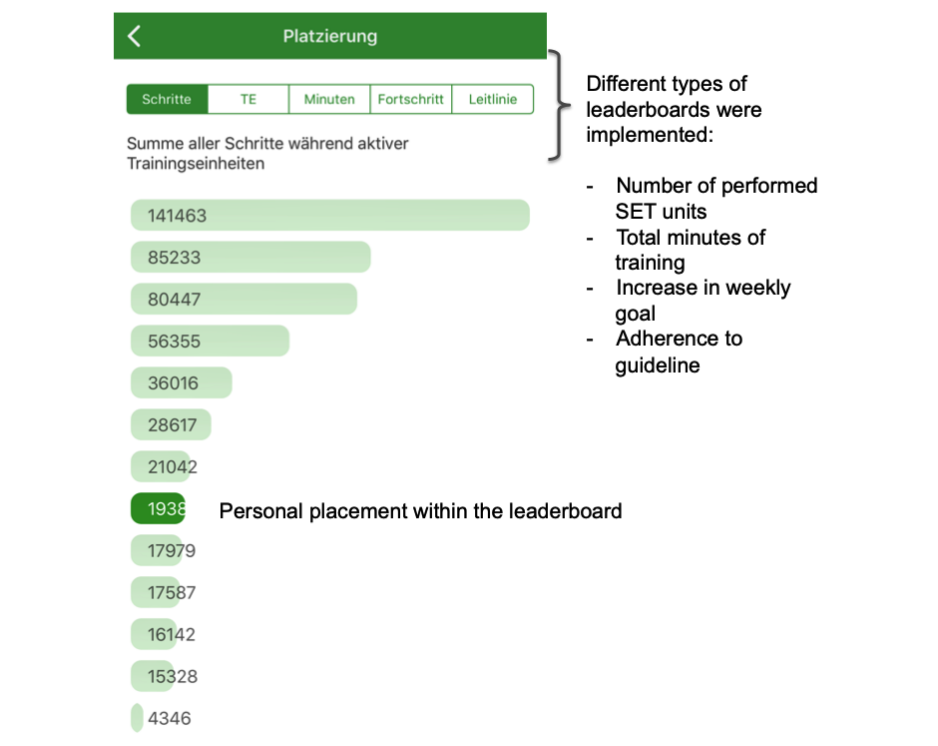
Leaderboard page. Different categories of leaderboards are included. Each gives the opportunity to improve the personal placement within the group. The evaluation process at the follow-up will bring further insights about which kind of leaderboard has the most impact regarding motivational aspects. SET: supervised exercise training.

TrackPAD was designed to cover the following requirements of PAD patients:

1. Weekly goal and self-evaluation of the training: At the beginning of each week, the app users are asked to set their weekly goal of SET units. On the basis of the completion rate of user’s SET units during the previous week, the app suggests a new weekly goal using an internal algorithm. The number of performed weekly SET units is not limited and can exceed the previously chosen weekly goal. In case of reaching or excelling the weekly goal, the app recommends to add 1 SET unit to the following weekly goal. In case of missing the weekly goal, the app recommends to reduce 1 unit the following week.
  As recommended by the guidelines, each unit includes 30 min of SET, but users can extend the duration of the unit. By taking a break, the units can also be split into intervals. TrackPAD records the number of intervals of a SET unit. Therefore, all breaks of running SET units because of pain or exhaustion are captured. To continue a SET unit, the user’s feedback is required. This feedback contains an assessment of each interval, regarding the pain level, breathing, and overall exhaustion.
2. Claudication reminder: After starting a new SET unit, a claudication reminder pops up (Figure 2), which needs to be confirmed actively. The user is reminded to adapt the own walking pace, incline, or even take the stairs to provoke moderate claudication during the SET unit, aiming for an increase in the pain-free walking distance. A pause button is provided to pause the SET unit after reaching a certain level of claudication.
3. Personal achievements: Personal progress of each user is recorded and linked to unlock medals (Figure 3). Achievements are rewarded, for example, a markable increase in user’s physical activity, activity on public holidays, or successes such as a daily physical activity of more than 15 min.
4. Leaderboard: The leaderboard contains different categories, such as the number of steps in single training session, number of completed training sessions, total minutes of physical activity, and percentage increase in physical activity, and shows the individual placement within the group (Figure 4).
5. Patient events: Information on upcoming patient events of the Department of cardiology and vascular medicine are stored and quickly accessible via the main menu.
6. PAD‑frequently asked questions (FAQ): A FAQ section is included addressing frequent technical issues, important contact information, general training advises, and also instructions in case of increasing or new pain during the training.

TrackPAD contains a password-protected admin function that allows to access data of each patient in real time. These data include weekly/monthly statistics of patients’ walking distance, time to the occurrence of pain, and frequency of exercise (Table 1). After trackPAD is once successfully installed, there is no need for further maintenance to be done by the users themselves.

The trackPAD main screen (Figure 1) is kept simple and has the following 4 components:

1. The upper part of the screen summaries the personal status of the current week, including the completed SET units of the weekly goal. The progress is presented as percentage and visualized by a moving stick figure.
2. The center of the screen shows the completed time of an active SET unit. This part also summaries the completed SET units each day of the week, including duration.
3. The lower part of the screen includes a link to the earned achievements and the personal rankings within the group.
4. Through the link in the upper right corner, events for patients, FAQ, privacy statement, and imprint are directly accessible.

There was no active reminder by the study team regarding the use of the app. A contact (eg, in case of technical problems or in case of a standard clinical examination) was always made by the patients and recorded. Except for the technical support, there were no cointerventions.

### Sample Size Considerations

The primary study aim was designed (1) for the general feasibility aspect and (2) to gain information regarding preferences of PAD-patients and their individual requirements. On the basis of these results, further patient-centered adjustment for trackPAD is planned. Therefore, it was estimated that a sample size of 20 participants per study arm would be feasible in a 3-month follow-up pilot study. The achieved power was estimated to be low with 0.46 (*t* test; type of power analysis=post hoc; effect size *d*=0.50; alpha error probability=.05, group 1 sample size=20; group 2 sample size=20). To allow for missing data and loss to follow up, we aimed to recruit 23 to 25 participants per study arm.

### Ethics Approval and Consent to Participate

This study was approved by the local ethics committee of the University of Duisburg-Essen (18‑8355‑BO). Written informed consent was taken from each participant, before any study procedures, and contact information was delivered to each participant. Any changes will be communicated to the ethics committee. The pilot study started in the beginning of November 2018.

## Results

### Recruitment and Randomization

The pilot study was funded in May 2018 by the Stfitung Universitätsmedizin and we received the IRB approval by the beginning of November 2018 (18-8355-BO). The enrollment started by December 2018 and ended in January 2019. All participants were recruited by the time of submission of the study protocol. The data analysis will start by the end of July and results to publish are expected by August 2019.

The majority of the potential participants (n=51) were recruited via the vascular outpatient clinic. In addition, 14 interested persons answered the announcement in the local newspaper, resulting in a total of 65 potential, eligible participants. The recruitment process took 7 weeks and was finished by November 2018. A total of 47 of the 65 recruited participants met the inclusion criteria and were enrolled to attend the initial baseline. The main reason for noninclusion of the remaining 18 potential participants was the missing of a suitable mobile phone. The summary of reasons for exclusion is listed in Table 2. To this point, no further participants dropped out.

**Table 2:** Dropout and exclusion reasons of recruited participants at baseline.

Category	Reason	Occurrences, n
Technical reasons	No suitable mobile phone	9
Individual reasons	No show up	5
	Personal reason	1
Medical reasons	No peripheral arterial disease/not matching medical inclusion criteria	1
	Matching medical exclusion criteria	2
Total dropouts and exclusions		18

All enrolled participants (n=47) were randomized, which resulted in the following assignment after randomization: 25 participants were randomized to the control group and 22 participants to the study group. One patient dropped out because of personal reasons shortly after randomization, but before announcing the result to the participant. Therefore, the control group decreased to 24 participants.

**Figure 5:**
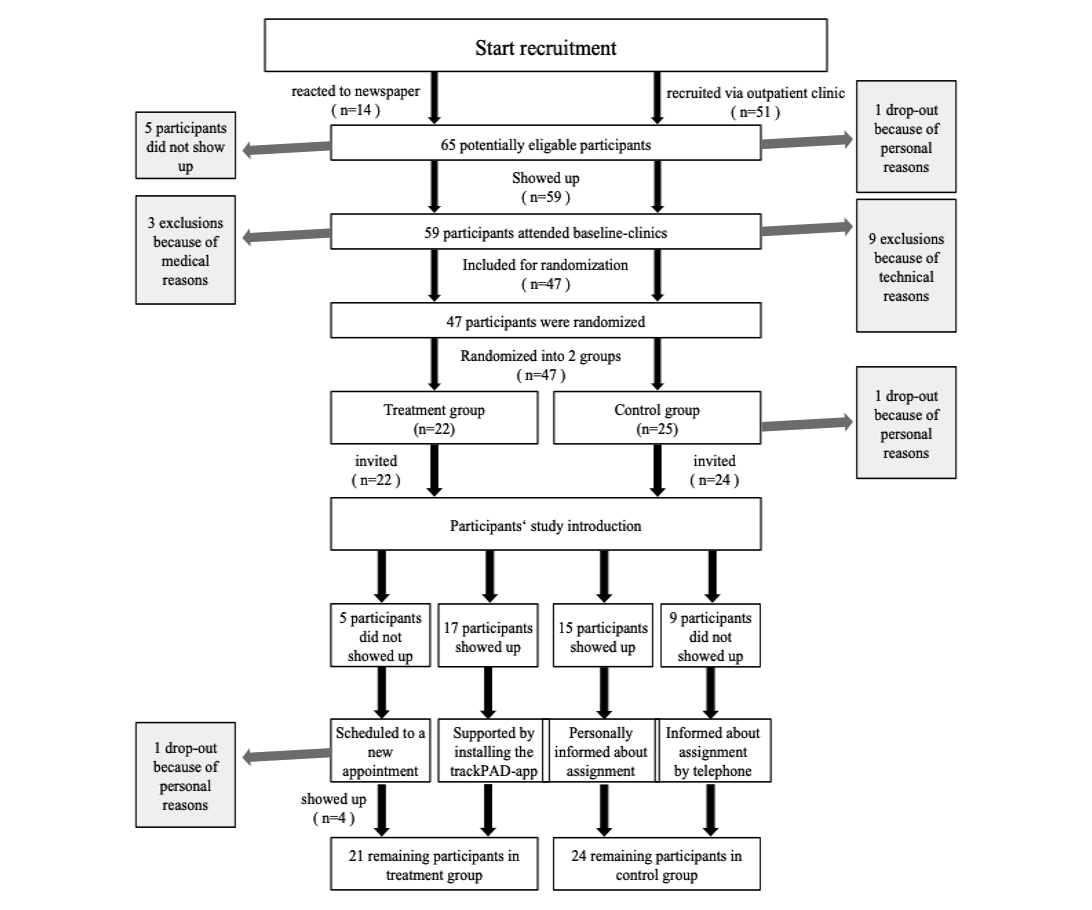
Quantitative development of screened patients until the beginning of trackPAD use. Reasons for dropouts and exclusions are shown.

All participants (n=46) were invited to a lecture for repeating the instructions for SET and receiving the personal result of the randomization. The study group remained after randomization for the download of trackPAD. A total of 32 of all 46 randomized participants showed up. All remaining participants (n=14) received their personal instruction including presentation, flyer, and the result of the randomization within the following 2 weeks. One dropout occurred in the study group after randomization due to technical problems. It was not replaced. Figure 5 summarizes the quantitative development of screened patients until the beginning of the trackPAD use, including dropouts and exclusions.

### Baseline

In total, 45 participants remained to take part in the pilot trial. The study group included 21 participants; the control group included 24 participants (Table 3). The mean age was 66.1 (SD 9.1) years and 44% (20/45) were male. The mean BMI was slightly elevated with 27.3 (SD 3.9) kg/m^2^. All participants performed the 6-min walk test with a mean baseline walking distance of 390.6m (SD 89.7) that was comparable between both groups (study group: mean 386.1m [SD 77.6] vs control group: mean 394.6m [SD 100.6]). The distance walked during the treadmill test was decreased compared with the 6-min walk test with 173.4m (SD 46.3), but also comparable between both groups (study group: mean 179.9m [SD 42.3] vs control group: mean 168.5m [SD 49.6]). It is to be noted that only 82% (37/45) of all participants were able to perform the treadmill test (study group 16/21 vs control group 21/24) because of instability or lack of balance on the treadmill.

**Table 3:** Summary of characteristics recorded at baseline.

Baseline characteristics		All participants (N=45)	Study group (N=21)	Control group (N=24)
Age (years), mean (SD)		66.1 (9.1)	65.3 (9.8)	66.9 (8.6)
Sex (male), n (%)		20 (44)	8 (38)	12 (50)
Body mass index (kg/m^2^), mean (SD)		27.3 (3.9)	27.3 (3.6)	27.3 (4.3)
**Walking distance**				
	6-min walk test (m), mean (SD)	390.6 (89.7)	386.1 (77.6)	394.6 (100.6)
	Treadmill test (m), mean (SD)	173.4 (46.3)	179.9 (42.3)	168.5 (49.6)
	Able to perform treadmill test, n (%)	37 (82)	16 (76)	21 (87)
**Ankle-brachial index**				
	Worst extremity, mean (SD)^b^	0.8 (0.2)	0.8 (0.2)	0.7 (0.20)
**Blood pressure (mm Hg),mean (SD)**				
	Systolic^b^	138.1 (22.5)	135.4 (26.9)	140.5 (17.9)
	Diastolic^b^	77.4 (13.0)	76.9 (13.4)	77.9 (13.0)
**Fontaine stage, n (%)**				
	Stage I	0 (0)	0 (0)	0 (0)
	Stage IIa	31 (69)	14 (67)	17 (71)
	Stage IIb	14 (31)	7 (33)	7 (29)
	Stage III	0 (0)	0 (0)	0 (0)
	Stage IV	0 (0)	0 (0)	0 (0)
**Level of weekly physical activity^a^**				
	Average number of active days, mean (SD)	2.2 (1.7)	2.2 (1.5)	2.0 (1.9)
	Weekly more than 30 min active, n (%)	24 (53)	12 (57)	22 (50)
**Comorbidities^a^, n (%)**				
	Myocardial infarction	8 (18)	3 (14)	5 (21)
	Heart failure	10 (22)	4 (19)	6 (25)
	Hypertension	37 (82)	17 (81)	20 (83)
	Stroke	30 (7)	10 (5)	2 (8)
	Diabetes mellitus	13 (29)	4 (19)	9 (38)
	Hypercholesterolemia	34 (76)	18 (86)	16 (67)
**Smoking (including e-cigarette)**				
	Within past 5 years, n (%)	38 (84)	17 (81)	21 (88)

^a^Characteristics based on participants’ information.

^b^All measures are before physical activity.

Most of the participants were former smokers (study group 13/21 vs control group 14/24), whereas 4 current smokers were in the study group and 7 in the control group.

## Discussion

### Principal Findings

As app stores are flooded with hundreds of fitness and activity apps, there is no app meeting the requirements for patients with PAD so far. The general increase in overall activity as provided by fitness apps or wearables is not equated with the execution of SET to improve the walking endurance among patients with PAD [17].

This pilot study is the first to evaluate an app prototype that was especially developed to support the implementation of SET in patients with PAD into everyday life. On the basis of the results of the evaluation process of the app prototype, a further patient-centered adjustment of trackPAD will follow. A patient-centered app development for this special patient collective is unique so far. Although some studies are recently published that deal with mHealth technologies (mainly via wearables and not as mobile phone-only solutions) and promote exercise in patients with PAD [16-18,20,33], these studies suffer from some limitations. Previous mobile interventions included apps and wearables off-the-rack, lacking tailored solution for this special patient collective and ignoring the fact of increasing SET performance rather than overall activity.

### Differentiation From Previous Studies

In the following text, we describe the main differences of the current trackPAD prototype compared with commonly used fitness apps.

1. Self-tracking of performed SET units and setting of weekly goal: Each unit has a minimum length of 30 min, as recommended by the current ESC guidelines on the diagnosis and treatment of PAD [26], and offers the opportunity to compare the personal weekly progress. A disadvantage of the current version is that weekly comparisons must be viewed manually. As part of the review following the pilot study, weekly status messages should pop-up automatically to reflect patients’ own progress and recommend achievable goals for the upcoming week. The reason to not include such an algorithm for the first time was the fact that PAD is a disease with high disabling potential and also affecting the functional status of capacity [34]. An automatic algorithm that is used in other common fitness apps does not seem to be feasible for this app.
2. Patient attention and empowerment: Each SET unit starts with a short reminder to reach the claudication. This note is important from our point of view as it often comes too short in the context of the activity and SET. The reminder function calls the importance of leaving the comfort zone instead of avoiding pain. Recent studies showed that patient education and empowerment through the increase of knowledge, skills, and confidence to overcome one’s disease [35] is associated with the willingness for health-related behavioral changes [36,37]. To strengthen the patients’ educational background, we included information regarding patient events on different medical topics. Future app development processes should also include a larger section for evidence-based health information regarding PAD and major risk factors.
3. Gamification aspects: Gamification uses game design elements combined with principles of psychology outside the gaming context as a strategy to promote a desired behavior or sustain healthy habits of subjects over time using Web-based behavioral interventions [38,39]. In the context of mHealth technologies, these game components can be used to entertain and also educate and motivate patients. Health behavior interventions can utilize gamification to deliver highly engaging content, enhancing the degree and depth of participant interaction and increasing behavior-change learning opportunities [38-40]. In this study, we used 2 major gamification elements to reward self-performance in terms of performed SET. The achievement of predefined personal goals unlocked medals in the medal mirror and served as digital reward. In addition, the ranking in the leaderboard provided a platform for competitive interaction between the participants. Both components, digital rewards and leaderboards, are common gamification features and were previously shown to have an influence on health-related behavior [41,42].

In addition to the pure feasibility, we examined whether there is an enhanced pain-free walking distance or other improvements in our treatment group, which is referred to trackPAD. Other possible improvements are a higher patient’s quality of life or a better leg perfusion resulting in reduced hospitalization, but they are limited to the 3-month follow-up period. The future vision of trackPAD is to serve as a tool for closing gaps in patient care owing to limited availability of personal and institutional resources.

Although SET is the basis of every PAD treatment, we limited the patient selection currently to Fontaine stage IIa or IIb to have a more homogeneous group in terms of walking distance and further stratification. Patients with Fontaine stage I are not limited by their walking distance and changes are hard to measure. Through the stratified randomization and dropouts, we did not receive an equal sized study and control group; however, as shown in Table 3, we currently see no reason for any potential bias caused by the distribution of this randomization. Nevertheless, the intention of this pilot study was to prove the feasibility of the upcoming main study and demonstrate potential pitfalls at an early stage. The average age of 66 years of the enrolled participants requires a highly intuitive app.

Although recent studies already investigated digital support tools on SET in patients with PAD [17,33,43], a stratification of walking distance was not performed yet. We advanced a first pilot study to access preliminary results for the inclusion into the calculation of the needed sample size.

The walking distance assessed by the 6‑min walk test was chosen as primary endpoint as the increase of 20m showed a minimal clinical importance in a recent publication [23]. The SD range assessed in the 6‑min walk test in patients with PAD was 51m to 69m.

### Limitations

One major limitation is surely the lack of blinding of the study participants. Motivational differences between the study and the control group might be driven by the fact that both groups were aware of the allocation to the respective group. A higher motivation to exercise because of the fact of only *having* the app or indeed *using* the app is not to differentiate. Further research is needed to address this issue. Owing to the focus on the first feasibility, this study is limited by its small sample size and its short follow-up period of only 3 months. As this paper focused on the technical development of an app especially designed for PAD-patients, it does not contain results beyond the baseline and also excludes a final app evaluation so far.

Another limitation is that although the patients were reminded via claudication reminder by starting each unit, no review of the actual activity load or walking pace until the onset of claudication was possible. Only an indirect assessment of claudication was available for the study group, including the number and duration of breaks within a SET unit and the pain assessment after each break, whereas no trend for SET performance in the control group is available. Moreover, in both study and control group, more than 50% of participants were already exercising and came from a certain exercise level assuming a simpler overcoming of motivational barriers. It is also to mention that studies including mobile interventions might serve as barriers to entry and the number of mobile phone possession in these patient groups is missing so far.

## Abbreviations

ABI: ankle-brachial index
ESC: European Society of Cardiology
FAQ: frequently asked questions
IC: intermittent claudication
mHealth: mobile health
PAD: peripheral arterial disease
SET: supervised exercise therapy

## Appendix

Multimedia Appendix 1 CONSORT-eHealth (V 1.6.1) checklist.
